# Generativity as a Route to Active Ageing

**DOI:** 10.1155/2012/647650

**Published:** 2012-08-07

**Authors:** Andreas Kruse, Eric Schmitt

**Affiliations:** Institute of Gerontology, Heidelberg University, 69115 Heidelberg, Germany

## Abstract

We elucidate the significance of active ageing from an individual as well as from a societal perspective. Taking an individual perspective, maintaining activity in later years is linked to successful ageing because of empirical relationships to positive self-perception, satisfaction with life, and development of competences, whereas from a societal perspective, active ageing implies usage of older people's life competences as a human capital of society—a societal imperative, particularly in times of demographic change but also more basically substantiated in an ethics of responsibility, intergenerational solidarity, and generation equity. We focus on the psychological construct of generativity which is interpreted as an aspect of the philosophical-anthropological category of joint responsibility. Our own research in Mexico and the Baltic States supports the notion that maintaining access to the public sphere and active engagement for others is a more basic individual concern than a life-stages specific developmental task. We report background and results of a Dialogue Forum Project Funding, a research cooperation between our institute and the Foundation Remembrance, Responsibility, and Future aimed to improve generativity in Belarus, Russia, and Ukraine by implementing and supporting local initiatives offering opportunities for intergenerational dialogue.

## 1. Active Ageing in Individual and Societal ****Perspective

Associating successful ageing with maintenance of activity has a long tradition in gerontology. Already in the 1960s, in the context of the classical controversy on propositions of disengagement theory, decreases in social roles and functions were interpreted as primarily reflecting prevalent misconceptions of old age and ageing, ageist stereotypes, and attitudes that contaminate external perception as well as self-conceptions and development of competences [1–4]. Although this pointed line of reasoning obviously neglects the significance of economic, political, and social structures as well as interindividual differences, the hypothesized relationships between role activity, self-concept, and satisfaction with life are still important for understanding positive or successful ageing [5, 6]. 

More recent gerontological theories elucidate that role activity in younger ages is a significant moderator of the relationship between older people's actual activity in specific social roles, satisfaction with these roles, and satisfaction with life. From the perspective of continuity theory [5] what predicts satisfaction and well-being is not the number of available roles and activities but the possibility to establish self-consistency via maintaining or substituting activities important for the self. Those older people who in younger ages identified themselves with specific social roles regularly benefit from continued role activity whereas others sometimes benefit from disengagement. Likewise, socioemotional selectivity theory [7, 8] elucidates that decreases in social contacts and social roles can have different outcomes depending on how older people succeed in satisfying individual motives for emotion regulation, identity, and information. Following this perspective the aforementioned motives change gradually over the lifespan. Because of a more limited future time perspective, emotional meaningful relationships become more and instrumental relationships become less important. Since people select among available relationships and activities, decreases in social roles sometimes reflect processes of optimization.

The modern understanding of active ageing is not least developed from a shift in research focus from questions of old age to questions of ageing. Human development is conceptualized as a lifelong process and a dynamic and continuous interplay of age-connected and age-independent developmental factors, with people explicitly conceived of as agents of own development. Taking a primarily individual perspective, modern concepts of active ageing can be understood as preventive concepts. Due to continuous engagement in personally meaningful relationships and contexts and systematic use of chances and opportunities, physical, psychological, and social losses and deficits can be prevented or at least substantially delayed. Referring to more recent definitions of productivity—that is, considering intellectual, emotional, and motivational expressions of productivity in higher age-groups [9–11]—it is further argued that even when suffering from severe physical losses and independency, people still have options to use capabilities and options to be productive for others or society as a whole [12]. Taking a primarily societal perspective, modern concepts of active ageing can be understood as means to use life competencies of the old as a human capital for society. Particularly in times of population ageing society's prosperity cannot be maintained alone by utilization of the potentials of younger people. As a consequence of an aging labor force, companies' competitiveness depends more and more on their ability to recognize, support, and effectively use older employees' potentials for innovation and creativity. Design of protective working environments, adjustment of working conditions, and offering opportunities for extended vocational training belong to the criteria of the companies' “demographic fitness” [13]. However, societal usage of life competencies in older people must be restricted to the area of work, paid or nonpaid.

With the term human capital we refer to the significance of life competencies for society and culture, that is, processes of initiating societal and cultural change and the extent to which societal and cultural change is determined by life competencies of the old. With the term life competencies we refer to experiences, strategies, and knowledge systems that people have acquired in earlier phases of the lifespan and through lifelong learning process [14–17]. Life competencies are built up in the context of effective coping and do enable people to maintain or reestablish a personal satisfying perspective on their life when confronted with serious problems, tasks, and challenges in later years. Building up life competencies in earlier years is a basic requirement for successful development in advanced age, that is, effective coping with challenges and demands of life in old age. Such challenges and demands include practical and psychological as well as interpersonal and ethical issues. Consequently, our understanding of life competencies is not limited to physical and cognitive strategies and knowledge systems acquired in the context of educational and occupational activities. Life competencies are also reflected in ethical judgments and voluntary activities in service for other people as well as in the willingness and readiness to take responsibility for oneself, for others, or for society. Empirical findings show that active coping with developmental tasks and the chances and limits of life can lead to the establishment of “expert knowledge” or “wisdom” with respect to questions of life [18].

Proceeding from a comprehensive understanding of productivity, several possibilities of leading an active and productive life in old age can be distinguished. Being interested in development, living conditions, and vital interests of younger people, the transmission of information to younger generations and the self-responsible reflection of experiences and knowledge systems of younger generations are examples for intellectual and emotional productivity in old age, since intergenerational discourses can initiate emotional and intellectual differentiation in older and younger participants. Moreover, by leading an independent and responsible life, even when confronted with serious problems or borderline situations, older people can give a good example of how to cope with problems and difficulties for younger people.

A good example for a productive intergenerational dialogue initiated by older people's coping processes can be found in a study on identity and life review in Jewish emigrants and extermination camp survivors by Kruse and Schmitt [19, 20]. In this study, one principal way of coping with stressful reminiscence—whereby stressful memories generally became more intense with older age—was based on an individual need to engage for others as well as for the society as a whole. This way of coping reflected an intense preoccupation with the future time perspective of younger people and a commitment to the development of the younger generation as well as to sociocultural and political issues with the aim to sensitize for the societal as well as the personal responsibility to the maintenance of democracy and the avoidance of fascism and anti-Semitism. The people who obviously run through these coping processes attended history or ethics lessons at school as “contemporary witnesses of history” and contributed to a responsible handling with history that way.

Realizing the potentials of a modern understanding of active ageing distinctly depends from intergenerational solidarity with older people's respective aspirations, motivations, and opportunities explicitly reflecting integrative potentials of a given society [21, 22]. Supporting active ageing is motivated by the superior ambition to substantiate a society for all ages. Consequently, the guiding principles of active ageing [23] explicitly include rights and obligations. Following the principle of subsidiarity [24], society is considered to be responsible to guarantee adequate opportunities to develop, expand, and realize potentials of old age; older people are considered to be obliged to use the opportunities offered by society to realize a self-responsible and jointly responsible life.

Asking for humans' basic responsibilities we proceed from coram structure as a figure of thought. The Latin coram can be translated with to keep in sight, the term coram public with in front of the public (the community, the society, the world). Proceeding from the aforementioned figure of thought, three spheres of human responsibility are differentiated which—taken together—enlighten the meaning of old age for individuals as well as for societies. The first sphere of responsibility is individual self-care, that is, the person's responsibility for and against oneself. The second sphere of responsibility is individuals' joint responsibility, that is, their willingness to engage for others and society. The third sphere of responsibility is individuals' obligation against God and creation, that is, joint responsibility for following generations, with those not yet been born included.

The concept of active ageing signifies a new understanding of ageing policy which—in distinction from a more traditional deficit- and welfare-oriented perspective—considers demographic change not as a basic problem but as a manageable challenge for societal development [25, 26]. As an answer to challenges of population ageing, the concept of active ageing refers to both an individual and a societal perspective: to actual and future resources and potentials of older people as well as to societal preconditions which have to be fulfilled for developing, expanding, and realizing potentials of older people.

Ageing policy is sometimes (mis)understood as social policy for older people, basically aimed to guarantee material security and health care. However, ageing policy is the entirety of measures focusing on maintaining or changing life situation of older people. Hence, questions of housing and transport as well as questions of education, employment, participation, and engagement are further important issues of ageing policy; ageing policy is a cross-section task since it is influenced by and has an impact on many other fields of policy: economic and financial policy, cultural and educational policy, and family policy. Emphasis of modern ageing policy is the basic requirement to offer older people equal chances for living a best possible independent and self-determined life within and for the respective society. Policy measures for older people should be inspired by the basic aim to establish a cultural context which allows for a competent and active ageing in the middle of the society and simultaneously can guarantee security and care for older people who suffer from impairment and disease. However, ageing policy must also compete with the task to deal adequately with needs, interests, and chances of younger and future generations. Supplies and rights in favor of the old as well as societal demands have to be reflected with regard to possible implications for the following generations. Aspects of generation equity are not only important for maintaining intergeneration contract—a basic cultural element of modern states—, but also for self concepts of older people. A positive impact on older people's self-concepts can be expected insofar as an intergenerational perspective enables them to find new sociocultural roles, that is, a basis for joint responsible living in old age. Equally important, that due to this perspective old age is increasingly integrated in the political and public sphere. To quote Arendt [27], the private dimension is enriched and expanded by the political dimension. 

## 2. Generativity

From both an individual as well as a societal perspective leading a good life in old age refers to social participation—in the words of political scientist Arendt [27] to an appropriate access to public sphere, that is, opportunities to establish and maintain social relationships, to engage for the fulfillment of interests and preferences of self and others, to take responsibility, and to actively contribute to further development of society. Most people experience respective opportunities as a source of belongingness, purpose and meaning in life, subjective well-being, and quality of life [19, 28, 29]. Individual's ability and motivation to empathize with other people, to engage for others, to contribute to the fulfillment of other people's needs, and to engage for society as a whole—joint responsibility [30]—are considered as a basic ethical category in old age.

In our own research we use the psychological construct of generativity which was described by Erik H. Erikson as the central topic of the seventh of eight psychosocial crises in lifelong development of ego identity and further developed by Dan McAdams, as an operationalization of the aforementioned ethical category.

According to Erikson [31], realization of generativity becomes an important developmental task in middle adulthood in the seventh of a total of eight psychosocial crises. Generativity can be defined as “concern in establishing and guiding the next generation” (page 267). As such, generativity is related to but also conceptually distinguished from the concepts of empathy, altruism, and intergenerational solidarity. In our understanding, the term empathy accentuates the “capacity to be affected by and share the emotional state of another”, whereas the term altruism refers to “behavior that benefits a recipient at a cost to the actor” [32], intergenerational solidarity can be defined in terms of “social cohesion between generations” [33]. Generativity can reflect individual needs, social norms, or both. Ideally, generative behavior proceeds from an empathic understanding of the needs, interests, and preferences of the younger generation. However, concerns for the next generation do not necessarily reflect the perspectives of younger people. Although generative behavior often implies older peoples' willingness to take costs for the benefit of others, engagement for younger generations can reflect selfish as well as altruistic motives. Basically, generativity is both motivated by intergenerational solidarity and contributing to maintaining and strengthening intergenerational ties. However, generative behavior is not always requested and accepted by younger people.

Already Erikson accentuated relatedness of the term to productivity and creativity, even though his understanding of generativity primarily focused on family relationships, particularly bearing and raising children. However, in his psychoanalytically inspired biographies of Erikson [34] and Mahatma Gandhi [35] already considered extra-familial realizations of generativity in the public sphere which might be regarded as the most productive and creative forms of generativity. Today, generativity is no longer understood as a concept “within” the individual but as a relational and multiply contextualized construct that links the person to the social world. 

Our understanding of generativity follows the conceptual and methodological framework provided by McAdams [36–38]. From this perspective there are two motivational sources of generativity, that is, cultural demand and inner desire. Cultural demand as a facet of generativity can be further explicated as reflecting age structure of society [39] and normative developmental expectations. In this context it should also be considered that cultural demand for generativity can substantially change over time, for example, against the background of demographic change interest in possibilities and preconditions of development and effective use of strengths and potentials of old age has grown worldwide. But generativity is not only prompted by society, not only societies have benefit from generative action. Inner desire as a second motivational source of generativity refers to two complementary basic human needs, that is, a “need to be needed”, to have meaningful relations to others, and a need for “symbolic immortality”, that is, to invest resources and potentials into things that outlive the self. The aforementioned motivational sources of generativity are reflected in two further facets of generativity, that is, a conscious concern for the next generation and a commitment to take responsibility for the next generation. The translation of concern and commitment into generative action depends on what has been described by Erikson [40, 41] as “belief in the species”, that is, “to place hope in the advancement and betterment of human life in succeeding generations, even in the face of strong evidence of human destructiveness and deprivation” [37]. Moreover, generativity is conceived of within the larger context of life-story theory of adult identity [36, 42]. From this perspective adults construct and try to live out a “generativity script” which not only reflects past generative action but is also important for current generative concerns and commitments as well as an understanding of what is worth to outlive the self and what can and should be transmitted to others to live on through generative efforts.

Generativity scripts are conceptualized as an important aspect of identity in higher age groups. In this context two aspects of identity become particularly important. (a) Identity—in the sense of an understanding a person has of himself and own development—is established in the context of narration. Adults define themselves and their position in society in terms of a life story that provides life with unity, purpose, and meaning [42–44]. Specific events and developments do not have an impact on individual identity in itself, instead they are selected from a magnitude of possible relevant events and developments (which moreover can be interpreted and evaluated in very different ways) and integrated into a coherent story (which starting from early adulthood regularly becomes a more and more definite story) which then builds the principal basis for understanding not only recent but also past events and developments. (b) Identity develops and becomes important in social interaction. Although referring to an individual understanding a person has of himself, identity in old age cannot be understood without considering social representations of old age and ageing, societal expectations and availability of social roles, and opportunity structures—for example, in the sense of a “generalized other” or a “Me” representing societal expectations and values [45]. As a consequence, processes of social change can have a profound impact on individual identity.

Understanding generativity as an important individual concern not only in middle adulthood but also in younger and particularly older age groups, the idea that generativity refers not only to an age-dependent developmental task but moreover to the philosophical-anthropological category of joint responsibility is supported by two larger international studies of our institute. 

In a comparative study we worked out together with colleagues from universities of Colima, Guadalayara, Juste and Madrid [46], we analysed relationships between generativity, optimism, and satisfaction with life in a sample of 3.308 subjects between 59 and 108 years of age, 1.506 from Mexico (394 from the region of Guadalayara, 387 from the region of Colima, 371 from the region of Armeria-Tecomán, and 354 from the region of Manzanillo), 1.200 from Spain (600 from the region of Alicante, 600 from the region of Extremadura), and 602 from Germany (region Heidelberg/Mannheim/Ludwigshafen). In each of the aforementioned 7 regions generativity was a highly significant predictor of optimism and satisfaction with life; regardless of the specific region considered, subjective health, financial resources, and family status could explain only for a much smaller amount of variance in optimism and satisfaction with life. These results support Veenhoven's model of the four qualities of life [47] which differentiates between “utility of life” (i.e., relevance for others) as a quality from “appreciation of life” (i.e., relevance for oneself), with these two qualities being independent predictors of subjective well-being. For all three countries no significant gender differences were found.

In an ongoing study in Estonia, Latvia, and Lithuania we analyze aspects of personal, social, and national identity, generativity, and perceptions of old age and ageing in three generations, that is, 15–25, 45–55, and 75–85 years old, in a sample of 360 subjects, 5 women and 5 men of each generation from each of the four biggest cities of Estonia (Tallinn, Tartu, Narva, and Kohtla-Järve), Latvia (Riga, Daugavpils, Liepaja, and Jelgava), and Lithuania (Vilnius, Kaunas, Klaipeda, and Siauliai). Results of this study show highly significant relationships between generativity, age stereotypes, and satisfaction with life, with generativity being an independent predictor of satisfaction with life after control for country, age, gender, national identity, and age stereotypes. In this study, women scored higher in generativity than men. Further analyses showed an interaction effect, indicating that the aforementioned gender effect is due to significant differences in the youngest age group. Our results on the relationship between gender and generativity are similar to those reported by McAdams and de St Aubin [36] for two US-American samples and support these authors hypothesis that “having children is more intimately linked with a man's generative concern than with a woman's (page 1008)”. Given the cross-sectional nature of the data, more research is needed on the topic whether lower generativity scores in younger men is a predictor, a consequence, or both of not yet having children.

Going beyond theoretical contributions of Erikson and McAdams we argue that generativity is an important concern not only in third but also in fourth age, sometimes increasing vulnerability might even trigger individual motives for generativity [38]. In the context of our research on quality of life in people suffering from dementia [48] we found evidence for generativity as an important individual concern in a substantial number of participants. Generativity concerns in these people became apparent particularly in reports about the disease to give closely related people insight into vulnerability to enable them to understand losses of control, challenging behaviors, and variation in emotions which care givers often attribute to inadequacy of own behavior, to give insight into processes of coping with border situations of human life, to inform about challenges, possibilities, and limits at the end of life, and more generally to contribute to a better understanding of people suffering from the disease.

## 3. Establishing Dialogue between Generations as a Strategy to Increase Generativity:  Basic Ideas and Results of an Intervention Project in Post-Soviet Societies

In the following we report results of an intervention study aimed to increase generativity in post-Soviet societies by implementing and supporting local initiatives offering opportunities for intergenerational dialogue. This research was worked out in the context of cooperation between the Institute of Gerontology of the University of Heidelberg and the Foundation “Remembrance, Responsibility and Future” started in 2008.

The Foundation “Remembrance, Responsibility, and Future” was established in 2000, primarily to make payments to former forced laborers. The payments programs were completed in 2007. The Foundation's capital of EUR 5.2 billion was provided by the German Government and German industry. A total of EUR 358 million was set aside as Foundation capital in order to finance project support. The Foundation finances its long-term funding activities out of the income generated by this capital. Work of the foundation can be subsumed under three principal activity areas and objectives:

- critical examination of history: anchoring the history of forced labor under National Socialism firmly in the European memory, communicating the life experience of the victims, promoting understanding of the different portrayals of history in Europe, and raising awareness of the Jewish contribution to European history,
- working for human rights: fostering commitment to democracy and human rights through history learning, initiating international projects that combat right-wing extremism, anti-Semitism, and modern forms of forced labor, which work to protect the victims, and developing capacity among the descendants of minority groups persecuted under National Socialism,
- commitment to the victims of National Socialism: engendering respect for the life histories of those persecuted under National Socialism and strengthening their involvement in society across generations, promoting willingness to help the victims at local and international level, and encouraging the development of models for providing humane support and care for the elderly.

Dialogue Forum Project Funding is a part of the third activity area “commitment to the victims of National Socialism.” In the context of cooperation in this activity area the Institute of Gerontology is primarily responsible for three tasks:

- supporting implementation and optimization of intergenerational projects in Belarus, Russia, and Ukraine to increase respect for victims of World War II, to strengthen people's involvement in society, and to contribute to adequate societal use of individual potentials and experiences,
- evaluating intergenerational projects to ensure that established intergenerational dialogues do not endanger individual and collective interests of the target group—for example, in terms of retraumatization or self-worth problems following from coping with aspects of own biography or transmission of knowledge and experiences,
- transmitting ideas and effects of intergenerational projects into national and international scientific and political discourse to contribute to both development of new and sustainability of already established models and ideas for intergenerational projects.

In the context of Dialog Forum Project Funding three aspects of identity were considered to be particularly important.

- Identity develops and becomes important in social interaction. Although referring to an individual understanding a person has of himself, identity in old age cannot be understood without considering social representations of old age and ageing, societal expectations and availability of social roles, and opportunity structures [14, 44, 49]—for example, in the sense of a “generalized other” [45], a “looking glass self” [50], or a “situational self” [35] representing societal expectations and values. As a consequence, processes of social change can have a profound impact on individual identity.
- Identity—in the sense of an understanding a person has of himself and own development—is established in the context of narration. Adults define themselves and their position in society in terms of a life story that provides life with unity, purpose, and meaning [42]. Specific events and developments do not have an impact on individual identity in itself, instead they are selected from a magnitude of possible relevant events and developments (which moreover can be interpreted and evaluated in very different ways) and integrated into a coherent story (which starting from early adulthood regularly becomes a more and more definite story) which then builds the principal basis for understanding not only recent but also past events and developments [51, 52].
- Such a narrative identity can be reconsidered or even revised in old age for several reasons. From the tradition of psychoanalysis it has been argued that defense mechanisms might lose effectiveness in old age; others have argued that age-related impairments and losses can force people to give up “protective illusions” [53, 54]. From a sociological perspective it can be argued that cohort flow necessarily implies somehow new perspectives on society [55], and changes in individual ageing processes as well as changes in societal age structures [56]. As a consequence, successful ageing necessarily depends on mutual exchange in intergenerational relationships. Moreover, societal change in post-Soviet societies since 1991 can be expected to have important implications for self-understanding in older people since collective representations of other countries—the former enemies that have been defeated in World War II—and history as well as basic political and economic orientations were subject to substantial change and revision. After the breakdown of the former Soviet Union particularly the younger generation is more oriented towards capitalistic values and a model of society which is represented best by the United States and Western European countries.
- Research on autobiographical memories suggests that events from adolescence and early adulthood are particularly important for narrative identity [57, 58]. However, intergenerational communication on such events is difficult in times of rapid social and political change.

Referring to the primary target group of intergenerational projects (i.e., victims of World War II in post-Soviet countries), four more specific aspects of identity and generativity that reflect insights, experiences, and hypothesis we developed during numerous interviews with members of our target group should be considered.

- The fate of former prisoners of war and forced laborers is still not adequately represented in popular accounts of national history. Considering self-understanding of older people in post-Soviet countries narrative identity implies that atrocities of war, losses, suffering, and deprivation of basic needs have not been useless or meaningless because of successful fighting against fascist enemies. For a substantial part of the population in post-Soviet countries former prisoners of war and forced laborers do not only failed to contribute to fighting back the threat of National Socialism, as collaborators they are even responsible for a longer-lasting war.
- Before breakdown of the former Soviet Union, respect for the heroes of war was an essential part of collective consciousness of history and patriotism. Veterans regularly visited history lessons in school since it was considered important to transmit their individual experiences of war to younger generations. However, prisoners of war and forced laborers stand for experiences that cannot be easily integrated into collective representations of war.

The cooperation between our institute and the Foundation Remembrance, Responsibility, and Future proceeds from the idea that the breakdown of the Soviet Union in 1991 and the fifth and sixth expansion of the European Union in 2004 and 2007 were not only accompanied by a development towards nationalistic sovereignty, democracy, constitutionality, and market economy but that in former Eastern bloc states and Soviet republics, questions of national and cultural identity have also gained in importance, became (again) subject of public discourse, and most importantly can—and sometimes even must—be answered differently. In this context it should be noted that in the former Soviet republics the decades of membership in the Soviet Union can be reconstructed as a loss of national independence, times of occupation, deportation, forced displacement, and attempted elimination of traditional language and culture as well as a progress in societal development giving raise to continued identification. In contemporary Ukraine about 75 percent of the national population speaks Ukrainian as first or second language, most people in the South and East prefer to speak Russian, whereas in the western part of the country most people prefer to speak Ukrainian. In contemporary Latvia about 30 percent of the population belongs to a Russian minority, 300.000 out of a total of 2.000.000 inhabitants do not have Latvian citizenship since they settled in Latvia before 1991 and have not passed naturalization proceedings afterwards. These two examples elucidate that national and cultural identity currently is a subject of controversy which must be clarified in intergenerational discourse.

It was expected that establishing informal contexts for intergenerational dialogue could contribute to realization of potentials of active ageing—namely generativity—in older people because the respective discourses not only result in a strengthening of own identity and related motives for joint responsibility. Local intergenerational projects should also offer opportunities to engage in new forms of generativity and experiences of being needed, accepted, and appreciated by others.

In 2009, 40 projects were implemented in Belarus, Ukraine, and Russia. 14 of these projects were evaluated by the Institute of Gerontology of Heidelberg University in a longitudinal research design consisting of 4 measurement points during a period of 2 years [59]. The central concept of project evaluation was generativity as an important facet of lifelong identity development, particularly in the form of older people to take responsibility for younger generations.

In evaluating intergenerational projects we used a combination of semistructured biographical interviews and psychometric scales for measurement of generativity and specific aspects of satisfaction and well-being in a sample of older people who participated in intergenerational projects. Additionally we assessed perceptions of generativity in a sample of younger project participants [59].

Concerning identity development, results indicate improvements in egointegrity, that is, older subjects' awareness of self-sameness and continuity in life and ability to accept one's life as a whole, including lost opportunities and unfulfilled aspirations and expectations [40]. Scales for measurement of self-acceptance [60], purpose in life [60], and meaningfulness [61] as well as assessment of attitudes towards own ageing and lonely dissatisfaction [62] showed highly significant increases. Moreover, analyses of semistructured interviews showed that both younger and older people perceived intergenerational dialogue as a fruitful input for self-understanding, and national and cultural identity. Younger people stated that during the funding period they developed a deeper understanding of national history and contemporary culture. Besides feelings of mutual acceptance and appreciation, older people stated to be increasingly able to understand values, interests, and preferences in younger generations, continuity and change in societal development, and similarities and differences between subsequent generations.

In all three countries we observed substantial increases in scores on Loyola Generativity Scale (LGS, [37]) for both older and younger subjects. As hypothesized from our theoretical-conceptual understanding of generativity, older subjects' improvements in generativity were substantially correlated with self-acceptance, purpose in life, meaningfulness, lonely dissatisfaction, and attitudes towards own ageing. 

At the first time of measurement LGS-scores for Ukrainian and Belarusian subjects were significantly higher than for Russian subjects. Improvement during the funding period was the highest in Ukraine. In Belarus and Russia we observed similar improvement; at measurement point 4 LGS-scores for participants from Russia approximated initial scores for participants from Belarus and Ukraine (see, [59, 63]). The aforementioned results support our hypothesis that societal demand for generativity might be particularly high when aspects of national and cultural identity are nationwide subject of controversy. This point elucidates the societal significance of potentials of older people in times of rapid social change.

In all three countries improvements in generativity are predicted by lower initial levels of generativity. Those who at least from a theoretical perspective of lifelong development are most in need of adequate intervention measures had the highest benefit from participating in intergenerational projects. Moreover, younger people perceived generativity in older people to be higher than generativity in their own generation—in our ongoing study in the Baltic states we find additional support for the hypothesis that younger people in post-Soviet societies explicitly appreciate (and do not neglect) knowledge and experiences as an important potential of old age. The latter result suggests that even if people of different generations have different perspectives on history and society there is no insurmountable generational gap. Overall, findings suggest that establishing dialogues between generations in the context of local projects is a promising measure to stimulate informal learning [15], to enhance generativity in both older and younger people, and to improve perceptions of older people's strengths and potentials in younger generations.

## 4. Concluding Remarks

In our view, the reported results of our research elucidate the societal obligation to shape public sphere on behalf of both older and younger generation in a way that—quoting Arendt [27]—maximizes opportunities for intergenerational encounters considering diversity, stimulating reflection of perspectives, discourse, and combined action, thereby enabling people to start something new—and this in the confidence to be recognized and accepted in one's peculiarity and to be appreciated for engagement for things and others. Establishing, maintaining, and strengthening of respective formal and informal context is considered a contribution to an age-friendly culture putting older people—their resources as well as their values, needs, and interests—in a similar way in the center of public sphere as younger people, enabling members of all generations leading lives in joint responsibility and social participation. Not only in Eastern Europe but also in Western countries, such a culture is still more a vision than reality, but nevertheless a basic imperative, not only in times of population ageing.

Development of an age-friendly culture depends on development of differentiated perceptions of old age and ageing. Older people need differentiated conceptions of old age and ageing for anticipation of developmental tasks, developing necessary resources, and using effectively existing resources in coping processes. Besides, also younger people benefit from differentiated conceptions of old age and ageing in terms of adaptive self-regulation, goal pursuit, and goal adjustment. Moreover, younger people need differentiated conceptions of old age and ageing for understanding and meeting adequately everyday interaction partners' specific limits, needs, strengths, and potentials.

As outlined in the 2nd International Plan of Action on Ageing [64], an age-friendly culture allowing for active ageing refers to building up a society for all ages. All ages have to be regarded as equal, not only with respect to rights but also with respect to obligations. Consequently, older people must not only claim their rights, rather they are—regardless if they are already retired or not—obliged against state and society. Considering demographic change, older people will have to take responsibility for further development of society. Societies are no longer able to relinquish the contribution of older people. Demographic change is irreversible. Whether European societies are able or not to grab the chance of using potentials of old age will be decisive for successfully meeting the challenges of globalization, structural change and international competition, and being prepared for necessities of reform and innovation to maintain prosperity.
